# Identification of *F3H*, Major Secondary Metabolite-Related Gene That Confers Resistance against Whitebacked Planthopper through QTL Mapping in Rice

**DOI:** 10.3390/plants10010081

**Published:** 2021-01-02

**Authors:** Eun-Gyeong Kim, Sopheap Yun, Jae-Ryoung Park, Kyung-Min Kim

**Affiliations:** 1 Division of Plant Biosciences, School of Applied Biosciences, College of Agriculture and Life Sciences, Kyungpook National University, Daegu 41566, Korea; dkqkxk632@naver.com (E.-G.K.); icd92@naver.com (J.-R.P.); 2 Coastal Agriculture Research Institute, Kyungpook National University, Daegu 41566, Korea; 3 Graduate School of Science, Royal University of Phnom Penh, Sangkat Teuk Laak 1, Russian Federation Boulevard, Toul Kork, Phnom Penh 12101, Cambodia; yunsopheap@gmail.com

**Keywords:** biotic stress, quantitative trait locus, rice, secondary metabolite, whitebacked planthopper

## Abstract

Whitebacked planthopper (WBPH) is a pest that causes serious damage to rice in Asian countries with a mild climate. WBPH causes severely rice yield losses and grain poor quality each year so needs biological control. Plants resist biotic and abiotic stress using expressing variety genes, such as kinase, phytohormones, transcription factors, and especially secondary metabolites. In this research, quantitative trait locus (QTL) mapping was performed by assigning the WBPH resistance score in the Cheongcheong/Nagdong doubled haploid (CNDH) line in 2018 and 2019. The RM280-RM6909 on chromosome 4 was detected as a duplicate in 2018, 2019, and derived from Cheongcheong. This region includes cell function, kinase, signaling, transcription factors, and secondary metabolites that protect plants from the stress of WBPH. The RM280-RM6909 on chromosome 4 contains candidate genes that are similar to the flavanone 3-hydroxylase (*F3H*) of rice. The *F3H* are homologous genes, which play an important role in biosynthesis defending against biotic stress in plants. After WBPH inoculation, the relative expression level of *F3H* was higher in resistant line than in a susceptible line. The newly identified WBPH resistance gene *F3H* by QTL mapping can be used for the breeding of rice cultivars that are resistant against WBPH.

## 1. Introduction

Whitebacked planthoppers (WBPH, *Sogatella furcifera*) cause serious damage to rice from long-distance migratory pests that exist in rice cultivation areas around the world, including South Korea, and cause enormous economic losses due to reduced production. Rice is one of the world’s three largest crops and, including South Korea, is the most popular crop that two-thirds of the world’s population consumes in the main meal [1]. However, in the fields where rice is bred, many pests that damage rice is also distributed. In particular, WBPH acts as a vector for southern rice black-streaked dwarf virus (SRBSDV) and is the most destructive rice pest [2] that causes various damages such as wilting, blight, and lodging of rice [3,4]. WBPH is a flying pest that arrives in South Korea from late June to July every year in Southern China and Southeast Asia [5]. The amount of WBPH flying is more than 10 times higher than that of brown planthopper (BPH), and the largest WBPH time of occurrence in South Korea is from the end of July to the beginning of August, and the injury symptom is shown after the end of August [6]. Currently, in the case of South Korea, WBPH control depends on chemicals, but it is difficult to detect WBPH damage at an early stage. Since spraying of agrochemicals in a state where the degree of damage is clearly indicated already missed the optimal time for recovery of damage, it is difficult to control WBPH only with agrochemical control, so the development of WBPH resistant cultivar is required [7,8,9,10]. The incidence of WBPH has increased in China and Vietnam, and a new kind of SRBSDV has been discovered [11]. Previous attempts to understand WBPH resistance in rice confirmed many important genes such as *Wbph1*, *Wbph2*, *Wbph3*, *wbph4*, *Wbph5* [12], *Wbph6(t)* [13,14], *Wbph7(t)*, and *Wbph8(t)* [15].

In South Korea, Cheongcheong is resistant to WBPH, but it is an indica type cultivar. In order to develop a japonica-type resistant cultivar, it must be crossed with indica type rice. In general, WBPH resistance has been successfully introduced to indica cultivars, but reports of japonica cultivars breeding are very poor [16]. This is because the resistant genetic resources of WBPH are mostly derived from indica cultivars. In the process of introducing resistance genetic resources into japonica type rice through the remote cross, agriculturally inferior traits were introduced together, making it difficult to develop practical japonica type cultivars [17].

WBPH resistance is a quantitative trait associated with polygenes with diverse and complex biological properties. Molecular markers, along with high-density loci, have been widely used to find quantitative trait locus (QTL) for resistance to biological stress such as insects and pathogens in many plants [18]. There are insufficient reports of QTL related to WBPH resistance in rice [19]. Sogawa et al., 2003 [20] discovered a QTL associated with resistance and susceptibility based on the sucking response of WBPH in the ‘Zhaiyeqing8/Jingxi17’ doubled haploid (DH) lines. Yamasaki et al., 2003 [21] discovered antibiotics related QTLs based on WBPH oviposition reactions using recombinant inbreeding lines obtained from ‘Asominori/IR24’. QTL analysis can be used to identify loci associated with WBPH resistance and apply the Marker-assisted selection (MAS) system to select lines with WBPH resistance [22].

In this research, Cheongcheong (Indica) and Nagdong (Japonica) were crossed to breeding Cheongcheong/Nagdong doubled haploid (CNDH) rice in order to establish a MAS system for breeding WBPH resistant rice cultivar. The DH line was used to mapping and analyze key genes and QTLs for various agricultural traits [23,24,25]. The main purpose of this research is to identify WBPH resistance genes by analysis candidate genes that have the greatest effect on WBPH resistance in the CNDH line using QTL mapping. This WBPH resistance gene can improve resistance to unpredictable WBPH infection due to extreme climate change in the future and can be effectively used for breeding WBPH resistance rice cultivars.

## 2. Results

### 2.1. Analysis of QTLs Associated with WBPH Resistance

**Figure 1:**
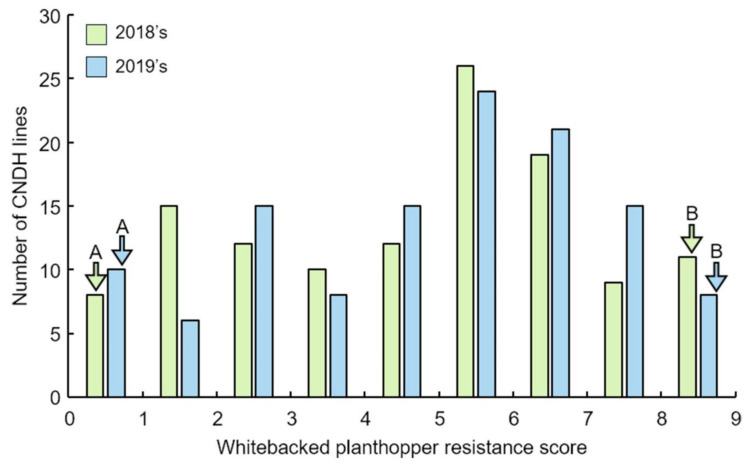
Frequency distribution based on the Whitebacked planthopper (WBPH) resistance score in the CNDH line. The WBPH resistance score showed normal distribution in the Cheongcheong/Nagdong doubled haploid (CNDH) line. Therefore, the response to WBPH resistance involved more than one gene. A, Cheongcheong. B, Nagdong.

**Figure 2:**
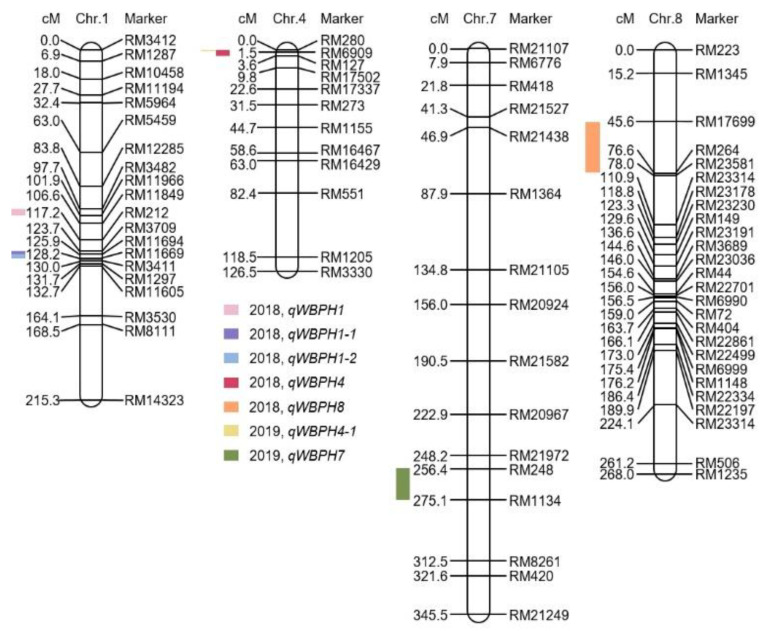
Chromosomal region of quantitative trait locus (QTL) associated with the WBPH resistance gene in the CNDH lines. QTL mapping was analyzed by assigning the WBPH resistance score in the Cheongcheong/Nagdong doubled haploid (CNDH) line in 2018, and 2019. WBPH resistance-related QTL mapping results indicated that the WBPH resistance gene was located on chromosomes 1, 4, 7, and 8. On chromosome 4, the RM280-RM127 was detected in 2018, and the RM280-RM6909 was detected in 2019.

The phenotypes of CNDH lines were measured to screen for resistant lines. The genetic map with an average of 10.6 cM between markers was constructed from 120 CNDH lines, which included 222 SSR (Simple Sequence Repeats) markers. The frequency distribution was created by assigning a resistance score after WBPH inoculation to 120 CNDH lines in 2018 and 2019 (Figure 1). The WBPH resistance score in the CNDH lines represented normal distributions. Therefore, WBPH resistance was a quantitative trait associated with more than one gene. After inoculating WBPH to Cheongcheong, Nagdong, and CNDH lines based on the resistance score, WBPH resistance-related QTL mapping was performed (Supplementary Table S1). As a result, the LOD value was 3.0 or more in seven QTLs. QTL was detected on chromosomes 1, 4, 7, and 8. WBPH resistance-related QTL mapping was performed in 2018 and 2019. In 2018, QTL was detected on chromosomes 1, 4, and 8; in 2019, QTL was detected on chromosomes 4 and 7. In 2018, *qWBPH1* was mapped at RM3482-RM11966 on chromosome 1 with an LOD value of 4.0 and a phenotypic change of 30%, *qWBPH1-1* was mapped at RM3709-RM11694 on chromosome 1 with an LOD value of 3.5 and a phenotypic change of 30%, *qWBPH1-2* was mapped at RM11694-RM11669 on chromosome 1 with an LOD value of 3.3 and a phenotypic change of 30%, *qWBPH4* was mapped at RM280-RM127 on chromosome 4 with an LOD value of 3.2 and a phenotypic change of 30%, and *qWBPH8* was mapped at RM17699-RM264 on chromosome 8 with an LOD value of 3.3 and a phenotypic change of 30%. In 2019, *qWBPH4-1* was mapped at RM280-RM6909 on chromosome 4 with an LOD value of 3.5 with a phenotypic change of 30% and *qWBPH7* was mapped at RM248-RM1134 on chromosome 7 with an LOD value of 3.0 and a phenotypic change of 30%. Of the WBPH resistance-related QTLs detected in 2018 and 2019, all were derived from Cheongcheong, except *qWBPH1*, which was derived from Nagdong. As a result of QTL mapping associated with WBPH resistance, RM280-RM6909 with an LOD value of 3.2 over on chromosome 4 was detected in duplicate for two years (Figure 2).

### 2.2. Candidate Gene Search Associated with WBPH Resistance Based on QTL Mapping

**Figure 3:**
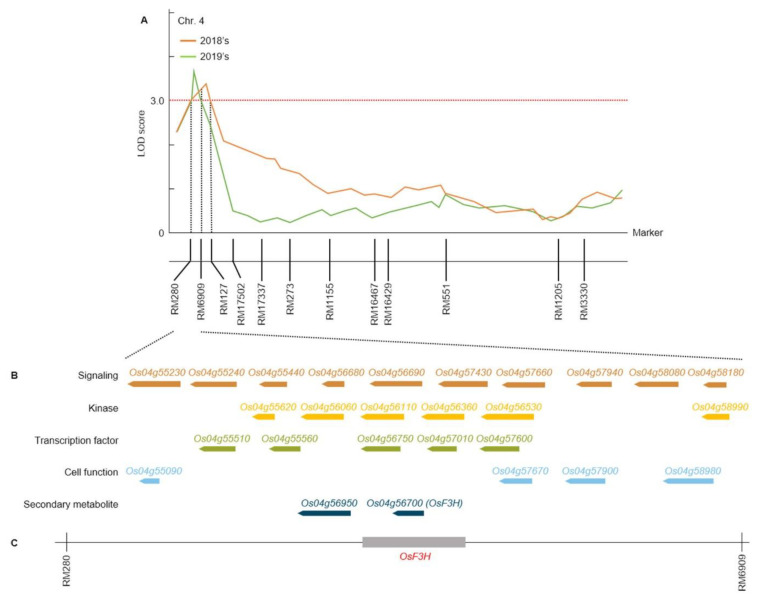
QTL mapping analysis based on WBPH resistance score in the CNDH lines. QTL mapping related to WBPH resistance was performed in 2018 and 2019. (**A**) RM280-RM6909 with an LOD value of 3.2 over on chromosome 4 was detected in duplicate for 2 years. (**B**) As a result of the physical map analysis of this region, candidate genes for signaling, kinase, transcription factor, cell function, and secondary metabolite involved in WBPH resistance was contained. (**C**) Among these candidate genes, *F3H*, a secondary metabolite, was identified.

We investigated candidate genes associated with WBPH resistance by selecting RM280-RM6909 on chromosome 4, which was detected in duplicate as a result of QTL mapping associated with WBPH resistance in 2018 and 2019. Twenty-seven open reading frames (ORFs) associated with WBPH resistance were identified using RiceXPro and the RAP-DB (https://rapdb.dna.affrc.go.jp). RM280-RM6909 on chromosome 4 contained candidate genes that correspond to cell function, kinase, signaling, secondary metabolites, and transcription factor associated with WBPH resistance (Figure 3, Supplementary Table S2).

### 2.3. Comparative Analysis of the Selection of Candidate Genes for WBPH Resistance

**Figure 4:**
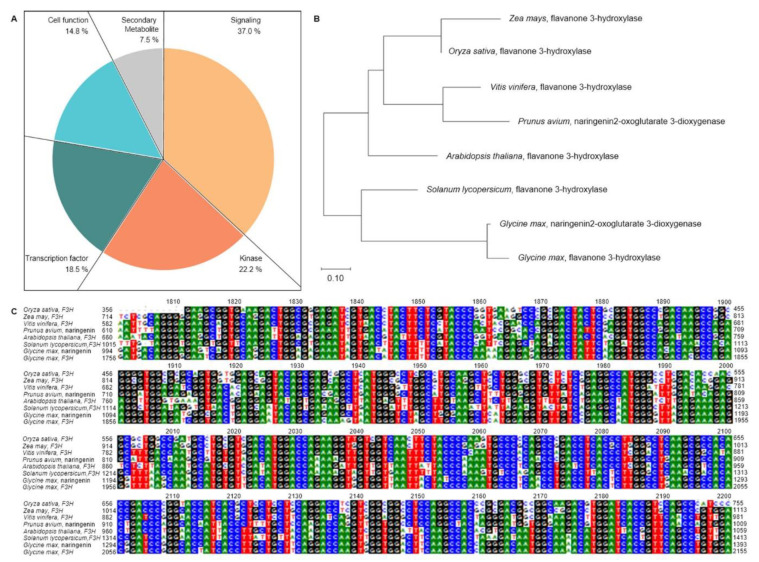
Candidate gene distribution based on WBPH resistance-related QTL mapping phylogenetic tree of *F3H*. (**A**) Candidate genes associated with WBPH resistance included cell function, kinase, signaling, transcription factors, and secondary metabolites. Signaling was 37.0%, kinase was 22.2%, transcription factors were 18.5%, cell function was 14.8%, and secondary metabolites were 7.5%. (**B**) Comparison of homology between *F3H* of *Oryza sativa* (*O. sativa*) and *F3H* of *A. thaliana*, *Z. mays*, *G. max*, *P. avium*, *S. lycopersicum*, and *V. vinifera*. *F3H* of *O. sativa* was the most similar to *F3H* of *Z. mays* and showed a relatively high similarity to *V. vinifera* and *P. avium*. (**C**) Multiple sequence alignments of *F3H*. Comparison to the conserved nucleic acid sequences found in the *F3H* domain region of *O. sativa*, *A. thaliana*, *Z. mays*, *G. max*, *P. avium*, *S. lycopersicum*, and *V. vinifera.*

Of the 27 ORFs, 37.0% were signaling, 22.2% were kinases, 18.5% were transcription factors, 14.8% were cell function, and 7.5% were secondary metabolites (Figure 4A). Among these candidate genes, *LOC_Os04g56700* has a similar function to flavanone 3-hydroxylase (*F3H*) in rice and acts as an important enzyme for flavonoid biosynthesis. Flavonoids are major secondary metabolites of plants and play important roles in defense mechanisms in plants. Because the *F3H* overexpression in rice showed WBPH resistance, the *F3H* gene was selected [26].

### 2.4. Phylogenetic Tree and Homology Sequence Analysis of Candidate Genes

BLAST analysis of the WBPH resistance gene *F3H* showed a similar sequence to naringenin. *F3H* was present not only in *O. sativa* but also in *A. thaliana*, *Glycine max*, and *Zea mays* as well as in *Prunus avium*, *Solanum lycopersicum*, and *Vitis vinifera*, which have high flavonoid contents. A phylogenetic tree was created to confirm the genetic similarity between the *F3H* and naringenin and the *F3H* present in rice (Figure 4B). The *F3H* of *O. sativa* was classified into the same group as *F3H* in *Z. mays* and had the highest homology. The *F3H* of *V. vinifera* and naringenin *2-oxoglutarate 3-dioxygenase* of *P. avium* were also grouped and had a relatively high homology (Figure 4C). These *F3H* genes had homologous domain regions.

### 2.5. Relative Expression Levels with Plant Defense Genes

**Figure 5:**
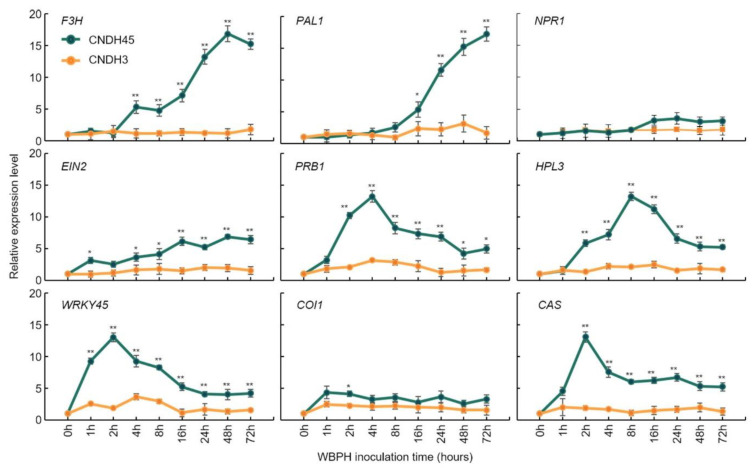
Comparison of the relative expression levels of *F3H* depending on the WBPH inoculation time in WBPH-resistant and WBPH-susceptible lines. *F3H* revealed by WBPH resistance-related QTL mapping was compared to the relative expression levels of WBPH resistance genes in *O. sativa* and *A. thaliana*. All genes were highly expressed in the WBPH-resistant line than in the WBPH-susceptible line. *F3H* showed a significant difference between the resistant and susceptible lines 4 h after WBPH inoculation (*p* = 0.01) and showed higher gene expression levels over time. CNDH45, WBPH-resistant line; CNDH3, WBPH-susceptible line. * indicates a significant difference at *p* < 0.05; ** indicates a significant difference at *p* < 0.01.

After inoculating WBPH to CNDH45 (WBPH-resistant line) and CNDH3 (WBPH-susceptible line), the relative expression level of *F3H* [27] was measured on certain time points. Moreover, plant defense genes of *O. sativa* and *Arabidopsis thaliana*, such as *phenylalanine ammonia-lyase* (*PAL1*) [28], *non-expressor of pathogenesis-related genes 1* (*NPR1 )* [29], *ethylene insensitive 2* (*EIN2)* [30], *pathogenesis-related protein* (*PRB1)* [31], hydroperoxide lyase3 (*HPL3)* [32], *WRKY45* [33], *coronatine insensitive 1* (*COI1)* [34], and *beta-caryophyllene synthase* (*CAS)* [35], were compared in relative expression levels. These genes are resistant to biotic stress, such as insect wounding. The expression level of *F3H* in rice had a significant difference between the resistant and susceptible lines 4 h after WBPH inoculation (*p* = 0.01; Figure 5). The expression level continued to increase from 4 to 48 h after WBPH inoculation and decreased after 48 h. *PAL1* also showed a similar tendency as *F3H*. *PAL1* had a significant difference between the resistant and susceptible lines 16 h after WBPH inoculation (*p* = 0.05). The expression levels of *EIN2*, *PRB1*, *HPL3*, *WRKY45*, *COI1*, and *CAS* also had a difference between the resistant and susceptible lines. *EIN2* and *WRKY45* showed a significant difference 1 h after WBPH inoculation (*p* = 0.05 and 0.01, respectively). *PRB1*, *HPL3*, *COI1*, and *CAS* showed significant differences 2 h after WBPH inoculation (*p* = 0.01, 0.01, 0.05, and 0.01, respectively). However, *COI1* showed a significant difference only 2 h after WBPH inoculation (*p* = 0.05), and there was no significant difference after that.

## 3. Discussions

WBPH is a significant cause of biotic stress that seriously damage rice growth in Asia. In modern agriculture, a synthetic pest control agent is used to reduce pest damage. However, synthetic pest control agents cause environmental pollution and WBPH becomes resistant to these agents, resulting in ecosystem disruption. An eco-friendly alternative is to breed rice cultivars that confer WBPH resistance gene in rice. In this research, CNDH lines, which were developed through cross of Cheongcheong (WBPH-resistant cultivar) and Nagdong (WBPH-susceptible cultivar), were subjected to QTL mapping based on the WBPH resistance score in 2018 and 2019. The WBPH resistance score in the CNDH lines represented normal distributions. Therefore, WBPH resistance is a quantitative trait associated with more than one gene. As a result, seven QTLs on chromosomes 1, 4, 5, 7, and 8 were detected. Among them, RM280-RM6909 on chromosome 4 was detected in duplicate for 2 years with an LOD value of 3.2 over. The LOD values for 2018 and 2019 are 3.2 and 3.5 with a phenotypic change of 30%. Twenty-seven ORFs were identified in the RM280-RM6909 region of chromosome 4, where 37.0% were for signaling, 22.2% for kinases, 18.5% for transcription factors, 14.8% for cell function, and 7.5% for secondary metabolites. Plants have a mechanism for synthesizing defense substances in response to invading pathogens. Recent research on plant-pathogen systems has accelerated. In particular, research on the mechanism for synthesizing plant secondary metabolites in response to biotic stress, such as pathogen inoculation, has emerged in plant biology [36]. Therefore, secondary metabolites produced in response to pathogen inoculation were selected as an available substance for an eco-friendly pest control agent [37]. Plant secondary metabolites mainly act on other species and influence ecological interactions with the plant and the environment [38]. Of the secondary metabolites, flavonoids occur widely in plants and can be divided into subgroups, including anthocyanidins, flavonols, flavones, flavanones, chalcones, dihydrochalcones, and dehydroflavonols. Thus, they are biologically significant and chemically diverse. Flavonoids not only induce the activity of medicinal plants and have a pharmacological effect [39,40,41,42] but also are synthesized through physiologically active compounds of the plants themselves, stress protectors, attractants, or appetite suppressants. Pathogens of various types play a significant role in plant resistance [36]. Many researches have already reported that the accumulation of flavonoids in plants makes them resistant to a variety of abiotic and biotic stresses [43,44]. Specifically, Brunetti et al., 2013 reported that flavonoids as effective in removing reactive oxygen species. Therefore, flavonoids are involved in the plant defense system through various actions. Moreover, Jan et al., 2020 [26] reported that the flavonoid series Quercetin, Delphinidin, Kaempferol, and Cyanidin increased in concentration by a significant difference at the 1% level in overexpression of *F3H* compared to control. Plants with increased flavonoids had increased resistance to WBPH compared to control. Therefore, an increase in flavonoids can confer resistance to biotic stress, including WBPH, and reduce damage.

In this research, as a result of QTL mapping associated with WBPH resistance, ORFs associated with secondary metabolites on chromosome 4 were detected. *LOC_Os04g56700* on chromosome 4 had a similar sequence to *F3H*. Some of the previously reported WBPH resistance genes compared to *LOC_Os04g56700* were WBPH resistance genes identified through QTL mapping, which confirmed their effects on WBPH resistance. *F3H* of rice had a similar effect to *PAL1*, which is an enzyme involved in the biosynthesis of polyphenolic compounds such as flavonoids [45]. Expression levels of *F3H* showed a significant difference between the resistant and susceptible line 4 h after WBPH inoculation (*p* = 0.01). These results proved that *F3H* identified in QTL mapping using the WBPH resistance score is precisely resistant to WBPH. The expression levels of *F3H* and *PAL1* increased significantly in the latter period of WBPH inoculation, and those of *PRB1*, *HPL3*, *WRKY45*, and *CAS* gradually decreased after the expression level increased in the early period after WBPH inoculation. *F3H* exists not only in *O. sativa* but also in *A. thaliana*, *G. max*, *P. avium*, *S. lycopersicum*, *V. vinifera*, and *Z. mays*. The homology of the *F3H* sequence was compared to *F3H* in other plants. As a result, *F3H* of *O. sativa* was classified into the same group as *F3H* of *Z. mays*, so it had the highest homology. *F3H* of *V. vinifera* and naringenin *2-oxoglutarate 3-dioxygenase* of *P. avium*, which have high flavonoid content, were also grouped, and had a relatively high homology. They have *F3H* and homology domains of rice, and it is possible to predict that they have similar functions. *F3H* is an enzyme involved in the biosynthetic pathway of dihydroflavonols in flavanones [46]. Dihydroflavonol synthesized by *F3H* is synthesized into leucocyanidin, which performs radical scavenging activities through *dihydroflavonol 4-reductase* (*DFR)* [47]. Leucocyanidin is synthesized by *anthocyanidin synthase* (*ANS*) into anthocyanidin, which prevents oxidative stress [48]. Finally, anthocyanin, which protects against invasion of bacteria and insects [49] and has excellent antioxidant effects, was synthesized by *UDP-glucose flavonoid 3-O-glucosyltransferase* (*UFGT)* [50,51].

## 4. Materials and Methods

### 4.1. Plant Materials and Treatments

The Cheongcheong/Nagdong double haploid (CNDH) line was bred in the field at Kyungpook National University from 2010. F_1_ obtained through crossing of Cheongcheong (Indica) and Nagdong (Japonica) was cultured to double haploid, and CNDH 120 line was developed. The CNDH line has been making generational progress for over 10 years and is currently being used as a bridging parent. In addition, it has been sufficiently used as a transforming and gene expression verification group also verified the stable expression of the gene using the CNDH line. The seeds were treated with a seed disinfectant in the dark at 25 °C for 4 days. The germinated seeds were sown on April 27, 2018 and April 26, 2019 at the experimental field at Kyungpook National University and transplanted on May 25, 2018 and May 24, 2019, with a planting distance of 30 × 15 cm. The amount of fertilizer applied was N–P_2_O_5_–K_2_O = 9.0–4.5–5.7 kg/10a, and the rice was cultivated according to the Rural Development Administration standard rice cultivation method.

### 4.2. WBPH Rearing

The rearing cage of WBPH was maintained at a temperature at 28 °C, humidity of 60%, and light intensity illumination of 16 h/day. Rice sowing was done weekly for feeding of WBPH and fresh seedlings of Chucheong was supplied for WBPH feeding. WBPH were able to move themselves to fresh plants. All WBPH had been reared in the oviposition stage.

### 4.3. Evaluation of WBPH Resistance in the CNDH Line

The WBPH resistance gene was evaluated using QTL mapping in WBPH-resistant and WBPH-susceptible lines. For bioassay analysis, seeds were sown in plastic trays (14 × 20 × 4.5 cm) at 3.5 × 4 cm intervals and cultivated to the seedling stage. Rice of the seedling stage was inoculated with second to third WBPH instars. About 15 WBPH were inoculated per plant. Phenotype change was observed after WBPH inoculation, and resistance scores were assigned based on plant damage evaluation. The evaluation of plant damage was performed using the Standard Evaluation System for Rice scale [52,53]. Resistance score was assigned 0 points if the plant was not damaged, 1 point if there were some damage, 3 points if the leaves were slightly underdeveloped, 5 points if the leaves were underdeveloped in more than half of the leaves, 7 points if more than half of the plants have died, and 9 points when the plant ultimately died. When Nagdong, a WBPH-susceptible cultivar, died, each plant was evaluated, and a resistance score of 9 was assigned.

### 4.4. QTLs Analysis of WBPH Resistance

QTL mapping was performed using WinQTL-cart2.5 [54]. The chromosome map of CNDH lines was created using the 222 SSR markers associated with WBPH resistance. Composite Interval Mapping was used to analyze WBPH resistance, and an LOD value of 3.0 or more was used to improve the accuracy of QTL mapping. The *R*^2^ value was used to represent the percentage of phenotypic changes that could be explained by QTL.

### 4.5. Identification of Candidate Genes through QTL Mapping

To identify candidate genes through QTL mapping, RiceXPro (https://ricexpro.dna.affrc.go.jp/) and Rapdb (https://rapdb.dna.affrc.go.jp/) were used. Candidate genes existing between markers obtained by QTL mapping were classified by function, and genes associated with WBPH resistance were analyzed.

### 4.6. Analysis of Expression Levels of Candidate Genes Resistant to WBPH

CNDH45 (WBPH-resistant line) and CNDH3 (WBPH-susceptible line) were inoculated with WBPH at the seedling stage when 3–4 main leaves were observed after sowing. The leaves were sampled at 0, 1, 2, 4, 8, 16, 24, 48, and 72 h after WBPH inoculation. RNA was extracted from the leaves using the RNeasy Plant Mini kit (QIAGEN, Hilden, Germany). cDNA was synthesized 80 ng RNA as a template using and qPCRBIO cDNA Synthesis kit (PCR Biosystems, Wayne, PA, USA). qRT-PCR was performed on the Eco Real-Time PCR System using WBPH resistance gene-specific primers (Supplementary Table S3). The qRT-PCR reaction contain 10 μL of 2× Real-time PCR Master Mix (BioFACT, Daejeon, Korea), 2 μL cDNA, 1 μL forward primer (10 pmol/μL), 1 μL reverse primer (10 pmol/μL), and DNase-free water to a final volume of 20 μL. The *OsActin* gene was used for the control. Each reaction was repeated three times, and the mean and standard deviation were represented.

### 4.7. Statistical Analysis

Data were analyzed by the SPSS program (IMMSPSS Statistics, version 22, IBMSPSS Statistics, version 22, Redmond, WC, USA). The mean and standard deviation were calculated and statistically analyzed through three repeated experiments. Furthermore, the candidate gene associated with WBPH resistance was compared to the control *OsActin* gene in terms of relative expression levels to analyze significant differences.

## 5. Conclusions

WBPH is a significant pest that causes severe damage to rice in Asian countries with a mild climate. Plants become resistant to biotic stress, such as WBPH, by synthesizing secondary metabolites to protect themselves. Therefore, secondary metabolites are essential elements in plant defense. QTL mapping was performed by assigning the WBPH resistance score in the CNDH line in 2018 and 2019. In the QTL mapping result for two years, the same region was mapped on chromosome 4. *F3H* was detected at the RM280-RM6909 region in rice. The *F3H* was involved in the synthesis of secondary metabolites. Moreover, this region was derived from Cheongcheong. Sequences similar to *F3H* have been found not only in *O. sativa* but also in *Z. mays* and have an essential role in biosynthesis defense against biotic stress in plants. They have homologous sequences, and *F3H* of *O. sativa* had the highest homology with *F3H* of *Z. mays*, so it was predicted that they would function similarly. After WBPH inoculation, the relative expression level of *F3H* was higher in the resistant line than in a susceptible line. The newly identified WBPH resistance gene *F3H* can be used for the development of rice cultivars that are resistant against WBPH, which has a negative impact on rice.

## Supplementary Materials

The following are available online at https://www.mdpi.com/2223-7747/10/1/81/s1. Table S1. QTLs associated with WBPH resistance in the CNDH lines. Table S2. Twenty-seven candidate genes identified between RM280 and RM6909 markers and their ORFs, which include various proteins associated with WBPH resistance. Table S3. WBPH resistance gene-specific primers for qRT-PCR.
