# Supplementary material for: Identification of F3H, Major Secondary Metabolite-Related Gene That Confers Resistance against Whitebacked Planthopper through QTL Mapping in Rice
Source: Plants (Basel). 2021 Jan 2;10(1):81. doi: 10.3390/plants10010081 (PMC7823371; doi:10.3390/plants10010081)
Supplement: Supplementary file 1 [file plants-10-00081-s001.pdf]

Supplementary Materials

Supplementary Table S1. QTLs associated with WBPH resistance in the CNDH populations.

Characteristics	Year	QTLs	Chromosome	Interval Markers[z]	LOD	Additive effect[y]	R^{2x}	Increasing effects[w]
Resistant score	2018	*qWBPH1*	1	RM3482-RM11966	4.0	-0.3	0.3	Nagdong
		qWBPH1-1	1	RM3709-RM11694	3.5	0.3	0.3	Cheongcheong
		qWBPH1-2	1	RM11694-RM11669	3.3	0.5	0.3	Cheongcheong
		qWBPH4	4	RM280-RM127	3.2	1.1	0.3	Cheongcheong
		qWBPH8	8	RM17699-RM264	3.3	0.7	0.3	Cheongcheong
	2019	*qWBPH4-1*	4	RM280-RM6909	3.5	1.1	0.3	Cheongcheong
		qWBPH7	7	RM248-RM1134	3.0	1.2	0.3	Cheongcheong

[z] Interval markers are those within the significance threshold on each border of the QTL range; [y] The proportion of evaluated phenotypic change attributable to a particular QTL was estimated using the coefficient of determination (R^2); [x] Positive values of the additive effect indicate that alleles from Cheongcheong are in the direction of increasing the traits; [w] Increase allele is the source of the allele that causes an increase in the measured trait.

Supplementary Table S2. Twenty-seven candidate genes identified between RM280 and RM6909 markers and their ORFs, which include various proteins associated with WBPH resistance.

**Function**	**Chr.**	**Gene locus**	**Description**
Cell function	4	*LOC_Os04g55090*	Protein prenyltransferase domain containing protein.
	4	*LOC_Os04g57670*	Protein prenyltransferase domain containing protein.
	4	*LOC_Os04g57900*	Protein prenyltransferase domain containing protein.
	4	*LOC_Os04g58980*	Protein prenyltransferase domain containing protein.
Kinase	4	*LOC_Os04g55620*	Protein kinase domain containing protein.
	4	*LOC_Os04g56060*	Protein kinase-like domain containing protein.
	4	*LOC_Os04g56110*	Protein kinase-like domain containing protein.
	4	*LOC_Os04g56360*	Protein kinase-like domain containing protein.
	4	*LOC_Os04g56530*	Protein kinase-like domain containing protein.
	4	*LOC_Os04g58990*	Protein kinase domain containing protein.
Signaling	4	*LOC_Os04g55230*	Tetratricopeptide-like helical domain containing protein.
	4	*LOC_Os04g55240*	VQ domain containing protein.
	4	*LOC_Os04g55440*	Leucine-rich repeat, plant specific containing protein.
	4	*LOC_Os04g56680*	Auxin responsive SAUR protein family protein.
	4	*LOC_Os04g56690*	Auxin responsive SAUR protein family protein.

	4	*LOC_Os04g57430*	Leucine-rich repeat, plant specific containing protein.
	4	*LOC_Os04g57660*	Leucine rich repeat, N-terminal domain containing protein.
	4	*LOC_Os04g57940*	Tetratricopeptide-like helical domain containing protein.
	4	*LOC_Os04g58080*	Leucine-rich repeat, plant specific containing protein.
	4	*LOC_Os04g58180*	WD40-like domain containing protein.
Secondary Metabolite	4	*LOC_Os04g56700*	Similar to Naringenin,2-oxoglutarate 3-dioxygenase (EC 1.14.11.9) (Flavanone 3- hydroxylase)
	4	*LOC_Os04g56950*	Similar to S-adenosyl-L-methionine:salicylic acid carboxyl methyltransferase-like protein.
Transcription factor	4	*LOC_Os04g55510*	Zinc finger, RING-type domain containing protein.
	4	*LOC_Os04g55560*	Pathogenesis-related transcriptional factor and ERF domain containing protein.
	4	*LOC_Os04g56750*	Zinc finger, CCCH-type domain containing protein.
	4	*LOC_Os04g57010*	Zinc finger, CCCH-type domain containing protein.
	4	*LOC_Os04g57600*	Zinc finger, CCCH-type domain containing protein.

Supplementary Table S3. WBPH resistance gene-specific primers for qRT-PCR.

Gene	Sequence (5' to 3')		GC contents (%)	Annealing Tm (℃)	Product size (bp)
F3H	Forward	TTCTCGTACCCGGTGAAGTC	55	59.5	84
	Reverse	GCTGTACTGCTCCACCACTG	60	59.5	
PAL1	Forward	TGCACGGTGGCAACTTCCAG	60	59.5	95
	Reverse	CTGCGCGAACATGAGCTTGC	60	59.5	
NPR1	Forward	CCGAGCTTTTGGATCTCGCAC	57	59.8	85
	Reverse	TTTAGGCTCTCTTCGCCTCGC	57	59.8	
EIN2	Forward	GCAGCTCCCTTGGCGTTTGA	60	59.5	89
	Reverse	CTTGCACTTGGGTTTGGGCTTTG	52	60.2	
PRB1	Forward	CGAGAACCTGTCGTGGAACC	60	59.5	91
	Reverse	GTAGTCGTAGGAGCCCTTCTC	57	59.8	
HPL3	Forward	CAGCAAGGAGGAGGCCATCA	60	59.5	88
	Reverse	GACCAGGAACGGCAGGAACA	60	59.5	
WRKY45	Forward	AGGACATGGAGAAGGGGAAGG	57	59.8	92
	Reverse	GAGAATCTGGTCGACGAAGCG	57	59.8	
COI1	Forward	GCTGCACCTCGTGATGGGC	68	61.4	92
	Reverse	TGAGCGCGTCGATGCGGTG	68	61.4	
CAS	Forward	ACGACAGGCAGGAGGCCAAT	60	59.5	93
	Reverse	CCAGCTCCATCTCCGCGAAAA	57	59.8	
